# Intravenous Administration of Umbilical Cord-Derived Mesenchymal Stem Cells (UC-MSC) for Acute Respiratory Distress Syndrome Due to COVID-19 Infection

**DOI:** 10.7759/cureus.44110

**Published:** 2023-08-25

**Authors:** Jesus A Perez, Juan J Parcero Valdes, Ruben Corral Moreno, Leonardo I Gomez Cuevas, Jose J Lopez, Thomas Ichim, Kristen McGreevy, Feng Lin, Santosh Kesari, Souvik Datta

**Affiliations:** 1 Medicine, Instituto de Medicina Regenerativa SA de CV, Tijuana, MEX; 2 Interventional Cardiology, Cruz Roja Tijuana, Tijuana, MEX; 3 Pulmonology, Cruz Roja Tijuana, Tijuana, MEX; 4 Critical Care, Cruz Roja Tijuana, Tijuana, MEX; 5 Neurology, Instituto de Medicina Regenerativa SA de CV, Tijuana, MEX; 6 Immunology, CureScience Institute, San Diego, USA; 7 Clinical Sciences, Pacific Neuroscience Institute, Santa Monica, USA; 8 Research and Development, CureScience Institute, San Diego, USA; 9 Translational Neurosciences, Pacific Neuroscience Institute, Santa Monica, USA; 10 Clinical Research, Rhenix Lifesciences, Hyderabad, IND

**Keywords:** immunomodulation, coronavirus, respiratory failure, ards, mesenchymal stem cells (mscs), covid 19

## Abstract

The COVID-19 pandemic has posed significant therapeutic challenges in addressing acute respiratory distress syndrome (ARDS). This serious illness has caused numerous fatalities worldwide and has had profound health and economic impacts. Previous studies have shown that mesenchymal stem cells (MSCs) can suppress ARDS. In this case series, we report on the treatment of nine patients with a single intravenous dose of 100 million hypoxic cultured umbilical cord-derived MSCs (UC-MSCs). Following the intravenous administration of UC-MSCs, obtained from the lining of the umbilical cord, longitudinal laboratory analysis revealed a sustained decrease in inflammatory markers and stabilized pulmonary function in eight out of nine patients. UC-MSCs possess immunomodulatory and anti-inflammatory properties, enabling them to attenuate the cytokine storm and potentially aid in lung repair. Importantly, no adverse events associated with the treatment were observed. These findings collectively suggest that a cell-based approach significantly enhances the survival rate of ARDS induced by SARS-CoV-2 and offers a promising treatment option in both preclinical and clinical settings.

## Introduction

Coronaviruses (CoVs) are a group of viruses known to cause respiratory or intestinal infections in both humans and animals. They were first identified in the 1960s from human embryonic tracheal organ cultures. Initially, only two species of human CoVs were recognized, but as of now, we have identified more than 10 different strains or variants of concern (VOCs) of CoVs, which are currently being monitored by the World Health Organization (WHO). There have been documented cases of human transmission of different coronaviruses, including severe acute respiratory syndrome coronavirus (SARS-CoV), which emerged in November 2002 in Guangdong Province, China, followed by Middle East respiratory syndrome coronavirus (MERS-CoV), discovered in June 2012 in Saudi Arabia. The most recent coronavirus to cause a global pandemic is the severe acute respiratory syndrome coronavirus 2 (SARS-CoV-2) [1], which was first identified in December 2019 in Wuhan, Hubei Province, China. The clinical presentation of SARS-CoV-2 infection can vary widely, ranging from asymptomatic shedding to severe respiratory infections in susceptible individuals [2]. Severe cases may require admission to the Intensive Care Unit (ICU) due to various complications, including shock requiring vasopressor therapy, acute respiratory distress syndrome (ARDS) potentially necessitating mechanical ventilation and multiorgan failure [3]. Currently, remdesivir (Veklury®) is the only fully FDA-approved therapeutic agent for the treatment of COVID-19 patients with severe consequences [4]. Additionally, two monoclonal antibodies, casirivimab and imdevimab, have been authorized under Emergency Use Authorization (EUA) by the FDA for combination treatment in mild to moderate COVID-19 patients, including adults and pediatric patients aged 12 years or older weighing at least 40 kilograms. Furthermore, an oral antiviral drug named molnupiravir has recently been approved by the U.K. Medicines and Healthcare Products Regulatory Agency (MHRA) [5].

The clinical course of hospitalized patients included admission to the Intensive Care Unit (25%) and mechanical ventilation support (10%). Among hospitalized patients, a significant portion required admission to the Intensive Care Unit (25%) and received mechanical ventilation support (10%). The dominant finding among critically ill patients is ARDS caused by profound acute hypoxemic respiratory failure [6, 7]. Common complications associated with COVID-19-related ARDS include acute kidney injury (AKI), pericardial effusion, cardiac injury, arrhythmia, and sudden cardiac death [8]. The typical clinical presentation of COVID-19 often involves symptoms such as fever, dry cough, fatigue, and myalgias. Other commonly reported symptoms include headache, sore throat, abdominal pain, and diarrhea [9]. Acute lung injury resulting from COVID-19 carries a high mortality rate of 30-40% and accounts for approximately 75,000 deaths annually, with a significantly higher impact on the elderly population [10].

Extensive studies have demonstrated that the initial stage of SARS-CoV-2 pathogenesis involves the recognition of the angiotensin-converting enzyme 2 (ACE 2) receptor by the virus's glycosylated spike protein [11, 12]. This receptor, known for its significant anti-inflammatory role, serves as the gateway for SARS-CoV-2, enabling the virus to bind more effectively, invade host cells, and propagate its infection [13]. Following the viral invasion, ACE2 cell surface expression is further diminished, leading to an upregulation of angiotensin II signaling within the lungs. This, in turn, triggers acute lung injury, characterized by impaired alveolar gas exchange, pulmonary edema, and the development of ARDS [14]. The virus's impact extends beyond the lungs, provoking a cascade of immune responses, including the release of pro-inflammatory cytokines such as IL-2, IL-6, IL-7, G-SCF, IP10, MCP1, MIP1A, and TNF-α. This excessive immune response, often referred to as a cytokine storm, can contribute to the development of complications such as colitis, myocarditis, sepsis, multiorgan failure, and potentially death [6]. Given the critical role of the cytokine storm in SARS-CoV-2 infection, mitigating its effects is crucial in the treatment of infected patients.

Mesenchymal stem cells (MSCs) are a type of multipotent stem cells derived from various sources such as bone marrow, umbilical cord, and other tissues, where they create a protective environment [15, 16]. In experimental models of organ injury, including stroke, dementia, myocardial infarction, acute kidney injury, liver cirrhosis, Crohn's disease, graft versus host disease, and lung injury, MSCs have been investigated as a preventive and therapeutic approach. They possess low immunogenicity and exhibit potent immunomodulatory effects, capable of attenuating or even preventing a cytokine storm, which positions them as a promising novel biological therapy for severe lung injury [16, 17]. By modulating immune functions, such as cell proliferation, cytokine production, and inhibiting T cell cytotoxicity, MSCs can influence the adaptive immune response through their impact on T cells. Studies have shown an increase in regulatory T cells in severely ill patients following MSCs administration, supporting their role in immune regulation and promoting localization in the alveolar spaces and airways [17]. Preclinical models of ARDS have demonstrated that MSCs possess the ability to migrate to injured lung sites, accompanied by increased production of angiotensin II [18]. The therapeutic benefits of MSCs involve the release of paracrine factors, including growth factors, to support the repair of damaged barriers caused by infection. They also enhance the host's defense against microbial infections by augmenting the activity of innate immune cells. Additionally, MSCs secrete growth factors that promote the restoration of alveolar epithelial fluid transport, involving both epithelial and endothelial growth factors [17]. When administered intravenously, MSCs can mitigate the severity of local and systemic inflammatory responses by releasing anti-inflammatory cytokines [18]. The anti-inflammatory properties of MSCs downregulate IL-6 expression, attenuate TNF-a concentration and elevate the expression of IL-10 concentration [17, 19]. As a result, lung inflammation and protein vessel permeability are reduced, leading to the prevention of pulmonary edema through the enhanced migration of MSCs [18].

## Materials and methods

Subject selection

This clinical study took place at the Red Cross Hospital in Tijuana, Mexico, spanning from April 2020 to July 2020. The study’s inclusion criteria were as follows: evidence of SARS-CoV-2 infection exhibited through Chest CT images or initial Chest radiographs showing multifocal bilateral airspace opacities upon admission, a positive quantitative reverse transcriptase-polymerase chain reaction (qRT-PCR) testing confirming SARS-CoV-2 infection, deteriorating pulmonary function associated with heightened oxygen needs, and increased levels of C-reactive protein (CRP), D-Dimer, and Ferritin. Written informed consent was obtained from all participants, and the study protocol (IMR-20-0415) was approved by the Institutional Review Boards (IRB).

MSCs treatment

Every participant in the study received a one-time intravenous infusion of 1 x 10^8^ MSCs derived from the umbilical cord lining. These cells were cultured under hypoxic conditions (5% O_2_) on Day 0.

Clinical and laboratory measurements

Study participants underwent daily monitoring, wherein their clinical data and vital signs were recorded. Specific laboratory analyses, focusing on inflammatory markers, were conducted on Days 0, 2, 4, and 14. Each patient provided an early morning fasting blood sample for investigations including: Lymphocyte subset counts (WBC, platelets, neutrophil and lymphocytes), hemoglobin, CRP, lactate dehydrogenase (LDH), D-dimer, and Ferritin. The collected serum was placed in separate plastic tubes, with some portions being stored at -70 degrees Celsius for future analysis. CT scan imaging was conducted both before the administration of the MSCs treatment and again about three weeks following the treatment.

## Results

A cohort of nine hospitalized patients with COVID-19, ranging in age from 46 to 83 years old, was included in this study. The characteristics of the patients are summarized in Table 1. Patients exhibited underlying medical conditions, including pulmonary hypertension, mixed cardiomyopathy, type 2 diabetes mellitus (DM2), hypertension, interstitial lung disease, and hepatitis C. Participants for this study were recruited from the Red Cross Hospital in Tijuana, Mexico. Inclusion criteria comprised of the following: presence of Chest CT images consistent with SARS-CoV-2 infection or initial Chest radiography displaying multifocal bilateral airspace opacities upon admission, confirmation of SARS-CoV-2 infection through positive quantitative reverse transcriptase-polymerase chain reaction (qRT-PCR) testing, deterioration of pulmonary function with increased oxygen requirements, as well as elevated levels of C-reactive protein, D-Dimer, and Ferritin.

**Table 1:** Characteristics of patients n: number; %: percentage; MSC: Mesenchymal stem cells; ARDS: Acute respiratory distress syndrome.

	All patients (n=9)
Median age – years (range)	68.8 (61 – 83)
Female sex – n (%)	4 (44.4)
Comorbid conditions	
Cardiovascular disease – n (%)	2 (22.2)
Asthma – n (%)	1 (11.1)
Hypertension – n (%)	5 (55.5)
Obesity – n (%)	4 (44.4)
Other pulmonary disease – n (%)	1 (11.1)
Type II Diabetes – n (%)	3 (33.3)
COVID-19 Severity on admission	
Mild – n (%)	2 (22.2)
Moderate – n (%)	3 (33.3)
Severe – n (%)	4 (44.4)
Days of admission prior to treatment with MSCs – median (range)	3 (0 – 4)
Failed or concurrent therapies	
Antibiotics, n (%)	9 (100)
Antiviral, n (%)	2 (22.2)
Anticoagulants, n (%)	8 (88.8)
Hydroxychloroquine, n (%)	7 (77.7)
Interleukin-6 inhibitor, n (%)	5 (55.5)
Steroids, n (%)	9 (100)
Oxygen therapy required, n (%)	
Mechanical ventilation, n (%)	4 (44.4)
Nasal O_2 _ n (%)	5 (55.5)
Pneumonia, n (%)	8 (88.8)
Concurrent bacterial pneumonia, n (%)	3 (33.3)
ARDS, n (%)	9 (100)
LOS post-treatment	11 (5 – 26)
Total length of stay (LOS) days	14 (6 – 26)
Survival – post-treatment with MSCs	
7 days, n (%)	8 (88.8)
14 days, n (%)	8 (88.8)
21 days, n (%)	8 (88.8)
30 days, n (%)	8 (88.8)

All patients in the study received a single intravenous infusion of 1 x 10^8^ umbilical cord lining-derived MSCs cultured under hypoxic conditions (5% O2) on Day 0. Daily monitoring of clinical data, vital signs, CT scans, and specific laboratory analyses (inflammatory markers) was performed on Days 0, 2, 4, and 14. Out of the nine treated patients, three required intubation and mechanical ventilation prior to receiving the stem cell infusion. Additionally, one patient was intubated three days after the stem cell infusion due to severe anxiety, inability to maintain the prone position, and CO_2_ retention resulting from sedative use. The clinical symptoms, treatment, and laboratory results of all patients can be found in Table 2.

**Table 2:** Clinical, treatment, and laboratory results of critically ill COVID-19 patients after admission RR: Respiratory rate; ARDS: Acute respiratory distress syndrome; HT: Hypertension; DM2: Type 2 diabetes mellitus.

Patient ID	001	002	003	004	005	006	007	008	009
Gender	F	F	M	F	M	M	M	M	F
Age	61	69	75	83	61	58	65	75	73
Days Hospitalized	9	26	6	19	6	25	10	12	19
Days in ICU	0	16	0	0	0	25	10	0	19
Days intubated	0	10	0	0	0	25	6	0	13
Symptoms. Day 0	Cough, dyspneic attacks, Sat 96% with 8 L/m O_2_, prone position, RR 14	Mechanical ventilation, prone position, abundant blood secretions, hypotension, bradycardia, Sat 97%, RR 20	Moderate respiratory distress, Sat 89% without supplementary oxygen, Sat 99% with 8 L/m O_2_, RR 27	Polypnea, Sat 96% with 15 L/m O_2_, hypotension, RR 23	Polypnea, Sat 96% with 15 L/m O_2_, RR 30	Mechanical ventilation, prone position, Sat 92%, intermittent tachycardia, RR 20	Mechanical ventilation, prone position, Sat 96%, intermittent tachycardia, RR 22	Tachypnea, moderate ARDS, Sat 95% with 3 L/m O_2_	Progressive dyspnoea, Persistent cough, Sat 92% with 15 L/m O_2_, RR 22
Symptoms. Day 4	Sat 100% with 5 L/m O_2_, RR 13	Mechanical ventilation, prone position, abundant secretions, bradycardia, Sat 93%, RR 18	No dyspnoea, no supplementary oxygen, Sat 96%, RR 18	Sat 94% with 15 L/m O_2_, intermittent prone position, RR 29	Sat 95% with 5 L/m O_2_, hypotension, RR 23	Mechanical ventilation, prone position, Sat 94%, tachycardia, RR 20	Mechanical ventilation, prone position, upper digestive hemorrhage, RR 20	Cough, no dyspnoea, Sat 93% with 10 L/m O_2_, intermittent prone position, RR 21	Severe dyspnoea, desaturation of 58%, Sat 92% with 60 L/m O_2_, intubation, RR 40
Reason for discharge	Overall improvement	Overall improvement	Overall improvement	Maximum clinical improvement, maximum benefit	Overall improvement	Hospitalized	Overall improvement	Maximum clinical improvement, maximum benefit	Overall improvement
Indications after discharge	None	Oxygen therapy, apixaban, Sulfamethoxazole and trimethoprim	Apixaban	Oxygen therapy	Oxygen therapy, apixaban	NA	Oxygen therapy	Oxygen therapy	NA
Relevant concomitant medical conditions	None	HT, Asthma	HT, DM2, ischemic cardiopathy	Severe pulmonary hypertension, interstitial lung disease	Hepatitis C reactivated 6 months ago	NA	DM2, HT	DM2, cardiomyopathy mixed, pacemaker	HT
Treatments received	Hydroxychloroquine, enoxaparin, baricitinib, azithromycin, steroids	Mechanical ventilation, tocilizumab, enoxaparin, steroids	Hydroxychloroquine, enoxaparin, baricitinib, azithromycin, steroids	Enoxaparin, baricitinib, tocilizumab, sildenafil, nifedipine, quetiapine	Hydroxychloroquine, enoxaparin, baricitinib, levofloxacin, enoxaparin	Mechanical ventilation, quetiapine, voriconazole, baricitinib, tocilizumab	Mechanical ventilation, sildenafil, baricitinib, steroids, enoxaparin	Methylprednisolone, enoxaparin, baricitinib, Hydroxychloroquine	Mechanical ventilation, enoxaparin, baricitinib, quetiapine, olanzapine
Clinical outcome	Good response	Tracheostomy, added bacterial pneumonia	Good response	High supplementary oxygen (50 L), torpid evolution, delirium	Good response	Tracheostomy, delirium	Controlled hemodynamic instability, upper digestive hemorrhage	Good response	Delirium

**Figure 1:**
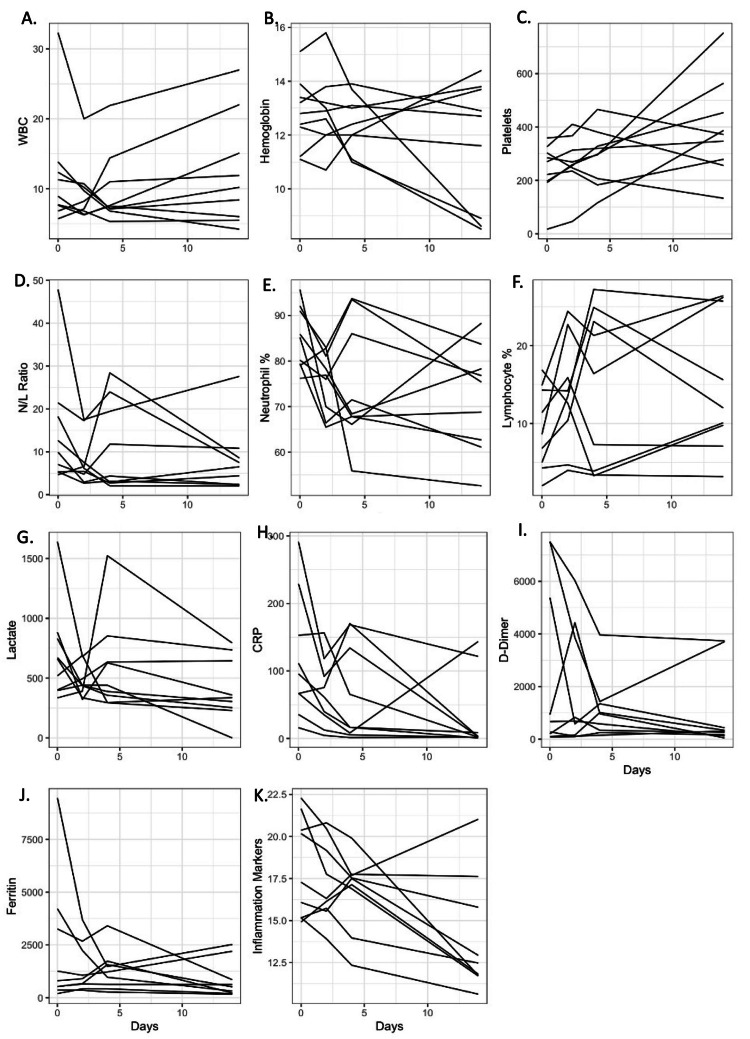
Changes of lymphocyte subset percentage, CRP, LDH, D-dimer, and Ferritin after MSC infusion. After MSC infusion, blood samples were drawn from patients on days 2, 4 and 14. Day of blood draws as control. Lymphocyte subset counts, CRP, LDH, D-dimer, and Ferritin were conducted in the clinical lab. (A) White blood cells (thousand/mm^3^); (B) Hemoglobin (g/dL); (C) Platelets (thousand/mm^3^); (D) N/L, Neutrophil - Lymphocyte ratio; (E) Neutrophil percentage; (F) Lymphocyte percentage; (G) Lactate (U/L); (H) C-reactive protein (mg/L); (I) D-dimer; (J) Ferritin (ng/ml); (K) Inflammation makers. CRP: C-reactive protein; LDH: Lactate dehydrogenase; MSC: Mesenchymal stem cells

Throughout the standard treatment course (which remained unchanged, with stem cells used primarily as an adjunct therapy), patients received a combination of medications, including hydroxychloroquine, tocilizumab, ivermectin, enoxaparin, baricitinib, and methylprednisolone. Prior to the administration of stem cells, three patients required full ventilatory support with high FiO_2_ (fraction of inspired oxygen) requirements. In each of these cases, after exhausting all known treatments and facing continued life-threatening consequences of the disease, the Mexican Health Authority (COFEPRIS) approved an emergency use expanded access investigational new drug application for umbilical cord lining-derived mesenchymal stem cells. On day 0 of the study, a total of 100 million cells were administered intravenously. Following the treatment, the levels of C-reactive protein (CRP), an acute phase protein commonly used as an early indicator of infectious or inflammatory conditions, showed a decline and remained stable (Figure 1H). The lymphocyte subset count and laboratory data of the patients are presented in Figure 1. Furthermore, the ratio of partial pressure of arterial oxygen (PaO_2_) to the percentage of inspired oxygen (FiO_2_), an important parameter used to monitor acute lung injury, demonstrated a steady increase after the stem cell infusion (data not shown). This positive trend suggests that the pulmonary alveoli were regaining their air-exchange function.

**Figure 2:**
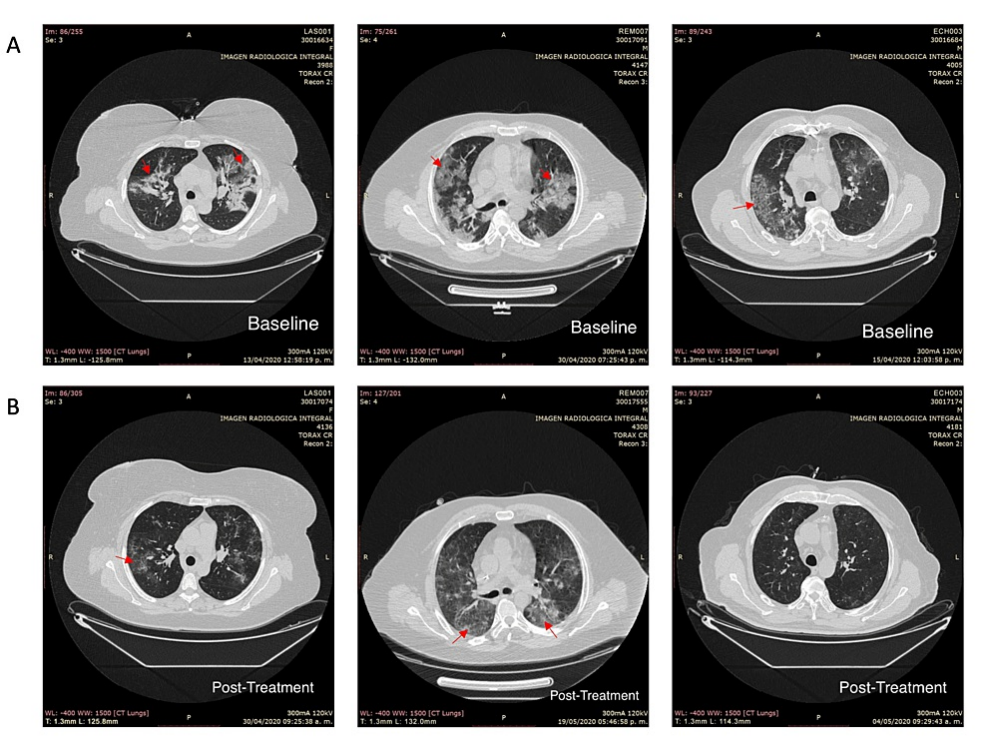
CT scan images of three patients. (A) Baseline chest imaging before MSC treatment. Images from left to right were patient number 001, 007, and 003. (B) Follow-up images after MSC treatment. Images from left to right were patient number 001, 007, and 003. MSC: Mesenchymal stem cells

Selected CT scan images of the patients are depicted in Figure 2. The initial two baseline chest images exhibit areas of consolidation and regions characterized by a “ground glass” appearance, highlighted in a mosaic “crazy paving” pattern (pointed out with arrows). These abnormalities involve approximately 25-50% of the parenchyma in both superior lobes and 50-75% in the middle and inferior lobes. The third reveals septal engrossment accompanied by a characteristic “ground glass” feature (also indicated with an arrow).

In the follow-up images, the first one demonstrates only “ground glass” opacities and septal thickening occupying 0-25% of either hemithorax. The second image shows consolidation zones primarily in the posterior segments, along with a predominant “ground glass” pattern (indicated with arrows) affecting approximately 25-50% of the parenchyma bilaterally. The third image exhibits discrete “ground glass” areas predominantly in the posterior segments, particularly on the left side (marked with an arrow). Overall, all the images indicate a notable improvement, characterized by a reduction in occupied air space and a significant decrease in consolidation.

As of day 26, after the infusion of MSCs, eight out of the nine patients who were enrolled in the study are still alive. Furthermore, these patients are demonstrating clinical improvement with minimal intervention required.

## Discussion

Our study reports encourage results following the administration of MSCs. This is highlighted by the immunomodulatory function of these cells, as evidenced by the consistent normalization of inflammatory biomarkers such as C-reactive protein, D-Dimer, and Ferritin. Furthermore, extensive post-infusion analyses revealed a significant decline in inflammatory cytokines and an increase in the population of cytokine-secreting immune cells, including CD3+, CD4+ T cells, CD8+ T cells, and CD19+ B cells, within 10 days in the circulating blood. These findings suggest that MSCs possess the ability to regulate the host immune response effectively. Additionally, MSCs exert anti-inflammatory and anti-apoptotic effects, thereby promoting alveolar fluid clearance and facilitating the repair of damaged pulmonary epithelial cells [20].

It has been previously reported that umbilical cord-derived mesenchymal stem cells have therapeutic efficacy in a model of acute lung injury induced by influenza A (H5N1) virus [21]. Building upon this knowledge, the utilization of hypoxic cultured cells for the treatment of COVID-19-induced ARDS represents a logical and promising approach. By controlling the oxygen pressure in vitro and reducing atmospheric oxygen from the standard 20% to lower physiological levels, the behavior of MSCs can be influenced. Hypoxic culture conditions have been found to inhibit cell senescence, promote cell proliferation, and enhance the stemness of MSCs [22]. Furthermore, hypoxia has been demonstrated to impact the paracrine effects of MSCs by modulating the secretion of various factors [23, 24]. The benefits of hypoxic culture preconditioning have also been observed in in vivo experiments, where hypoxic cultured MSCs exhibited superior performance after transplantation in ischemic environments [25]. Moreover, hypoxic cultured MSCs have demonstrated enhanced survival, migration, angiogenesis, and engraftment in various tissues within the body [26]. Based on these observations, we propose that when intravenously administered, hypoxic cultured umbilical-derived MSCs migrate to the lungs and exert positive effects on the pulmonary microenvironment. These effects include the protection of alveolar epithelial cells, prevention of pulmonary fibrosis, and improvement of lung function by suppressing or attenuating the cytokine storm through the secretion of potent anti-inflammatory factors, such as CXCL5 [27]. The combination of hypoxic culture and the therapeutic properties of umbilical cord-derived MSCs is particularly crucial in conditioning cells that are best suited for the targeted microenvironment of the lungs, thereby maximizing the therapeutic response in patients suffering from COVID-19.

However, our study has several limitations. These include a small sample size, lack of a control group, potential selection bias due to single-site recruitment, and the presence of multiple comorbidities and medications, which complicates interpretation. Additionally, a short follow-up period, possible subjectivity in CT scans interpretation, lack of blinding, and unaddressed confounding factors also limit the study's reliability. Moreover, we largely relied on short-term clinical improvement, which may not necessarily correspond to full recovery or long-term survival.

Currently, over 85 trials registered on ClinicalTrials.gov mention the use of MSCs in various therapeutic applications [28-30]. To the best of our knowledge, this is the first report to utilize hypoxic cultured umbilical-derived MSCs for the treatment of COVID-19-induced ARDS. Considering the urgency to control the pandemic and the pressing need for safe and effective therapeutic interventions, our data presents a potential option for treating critically ill patients under compassionate use protocols.

## Conclusions

Several therapies, including the FDA-approved remdesivir, as well as emergency-use authorized drugs such as tocilizumab and baricitinib, have shown efficacy in mitigating the severity of SARS-CoV-2 infection. However, these treatments are not without significant side effects and can be expensive. Additionally, monoclonal antibody therapies have been utilized, but their availability and cost limit their widespread use. As more is understood about the pathophysiology of SARS-CoV-2, researchers are actively exploring other potential drug candidates.

In this context, the clinical improvement observed in our patients, along with the progressive reduction and stabilization of inflammatory cytokines following MSC infusion, provides a strong rationale for further investigation into the use of MSCs for COVID-19-associated ARDS. We confirm that hypoxic cultured umbilical-derived mesenchymal stem cells can be administered to patients with COVID-19 without acute side effects. Moreover, these stem cells have the potential to contribute to the reduction of pro-inflammatory factors associated with the infectious process triggered by SARS-CoV-2. These preliminary findings align with the results reported by other researchers, highlighting the need for larger randomized, double-blinded, multi-center studies. Such studies will provide deeper insights into the role that MSC-based treatments can play in effectively managing this condition. By conducting rigorous research on a larger scale, we can better understand the safety and efficacy of MSC therapy, paving the way for its potential integration into standard treatment protocols for COVID-19.
